# Making sense of symptoms, clinicians and systems: a qualitative evaluation of a facilitated support group for patients with medically unexplained symptoms

**DOI:** 10.1186/s12875-021-01495-9

**Published:** 2021-07-01

**Authors:** Michelle Marcinow, Jane Sandercock, Chelsea D’Silva, David Daien, Carly Ellis, Christine Dias, Elizabeth Mansfield

**Affiliations:** 1 Institute for Better Health, Trillium Health Partners, Administrative Building – 6th Floor, 100 Queensway West, Mississauga, ON L5B 1B8 Canada; 2 Family Medicine, Institute for Better Health, Trillium Health Partners, 100 Queensway West, Mississauga, ON L5B 1B8 Canada; 3 Strategic Projects, Trillium Health Partners, 100 Queensway West, Mississauga, ON L5B 1B8 Canada; 4 Medical Psychiatry Alliance, Trillium Health Partners, 100 Queensway West, Mississauga, ON L5B 1B8 Canada; 5 Department of Occupational Health, University of Toronto, 6 Queen’s Park Crescent West, Toronto, ON M5S 3H2 Canada

**Keywords:** Qualitative research, Patient experience, Medically unexplained symptoms, Facilitated support groups, Self-management, Patient-provider communication

## Abstract

**Objectives:**

Health services to date have inadequately addressed the physical and mental health needs of patients with medically unexplained symptoms. This qualitative study evaluates a piloted facilitated support group (FSG) developed for patients with medically unexplained symptoms to inform recommendations and resources for this patient population.

**Methods:**

Using a qualitative descriptive design, we conducted and thematically analyzed semi-structured interviews with participants (n = 8) and facilitators (n = 4) to explore their experiences of the facilitated support group. Common themes that captured strengths and challenges of the facilitated support group were identified.

**Results:**

The following key themes were identified through analysis of the data: Participants described 1) feeling validated through sharing similar experiences with peers; 2) learning practical symptom management and coping strategies; and 3) gaining new perspectives for navigating conversations with PCPs.

**Conclusions:**

Our findings show that a facilitated support group may provide additional forms of support and resources for patients with medically unexplained symptoms, filling a gap in currently available clinical care offered by health care professionals. *Potential implications:* This paper highlights lessons learned that can inform the design and delivery of future supports and resources directed toward optimizing patient care for this underserved patient population. Our findings are relevant to those who are involved in direct patient care or involved in designing and implementing self-management programs.

**Supplementary Information:**

The online version contains supplementary material available at 10.1186/s12875-021-01495-9.

## Introduction

Medically unexplained symptoms (MUS) refers to the physical manifestations, such as chronic headaches, pain or fatigue that cannot be explained by an established medical diagnosis [1]. While some patients with MUS will recover after long-lasting and disabling symptoms [2], many others will continue experiencing MUS over time [3]. Individuals without a medical diagnosis and ‘socially accepted’ biomedical explanation must continuously defend and justify their illness to others [4]. Living with MUS can impact one’s quality of life due to associated anxiety [5, 6], depression [5], impaired social functioning [5], co-morbidities [7], the pressure of constantly making efforts to rationalize the illness [4], and living and coping with chronic symptoms (e.g., fatigue, pain) [8]. Having MUS is often overwhelming for patients and can limit their ability to engage in everyday activities, such as work [9], home life and maintaining social relationships [8]. In addition to creating stress on the individual, and often their support network, the burden of care associated with treating and investigating patients with MUS is significant. Compared to other patient populations, patients with MUS tend to be higher healthcare users [5–7, 10], have higher medical costs [11], and often receive unnecessary, and potentially harmful, tests, referrals, and/or treatments [12]. Due to the limitations of the biomedical model being oriented towards acute care rather than supporting chronic health conditions, this patient population continues to be underserved despite having more interactions with the healthcare system [13].

Previous research has highlighted that a patient-centered approach is needed when caring for patients with MUS [14]. Health care professionals should shift attention from “curing” to “caring and coping” [9, 15], listen to patients’ interpretations of their illness, care expectations and lived experiences [8, 16–18], and acknowledge that patient symptoms are real and not imagined [9, 15, 19]. However, the biomedical model typically used to address the needs of patients with MUS does not account for the complexity of patients’ health concerns [20, 21], lacks a patient-centered approach to care management [22], and offers physicians little formal training about how to approach and support this patient population [23]. Yet, both physicians and patients often rely on the biomedical model to explain MUS [24] and perceptions about what is real versus believed can lead to conflicts [25]. While some patients with MUS take comfort in having their symptoms recognized as part of an acknowledged condition [26], since this understanding can lead to specific treatments or self-management strategies [27], others perceive that a diagnosis and/or cure is unlikely and seek alternative support to cope with symptoms and increase quality of life [9].

Facilitated support groups (FSGs) (i.e., professionally facilitated patient/peer groups), are promoted as a way to help patients living with chronic conditions self-manage symptoms and improve their quality of life [8, 28–30]. These groups are often beneficial for patients because they can assist in managing and making sense of one’s illness and social identity [30, 31], improving functional ability [30, 32], and reducing the dependence on primary care physicians (PCPs) [32]. Because patients with MUS often fall outside the boundaries of standard medical care [4], it is difficult for patients with MUS to find supports, resources and/or connect with patients with similar narratives [4, 33]. Facilitated support groups have the ability to create a network of ongoing support that can have both physical and psychological benefits for individuals living with a chronic conditions, such as MUS [34]. However, there is a limited understanding of how supports beyond psychological therapy groups (e.g., cognitive behavioural therapy) can help patients with MUS navigate the challenges of daily living [8, 35]. This paper reports findings from an exploratory qualitative evaluation of a FSG developed for patients with MUS to answer the following question: How can a FSG provide support for patients navigating living with MUS?

## Methods

### Setting – facilitated support group

The FSG was developed by the pilot project team as one of many pilot projects supported by the Medical Psychiatry Alliance (MPA), a multi-year collaborative partnership involving three hospitals (community, psychiatric and paediatric) and a local university, dedicated to improving care for patients who suffer from both physical and psychiatric conditions*.* MPA initiatives focused on better integrating physical and mental health services for patients across the lifespan, many of whom who have previously been underserved by the healthcare system. To ensure the relevance of the FSG to the MUS patient community, the pilot project team held a series of patient engagement sessions to guide the development of the program’s content and structure. In these sessions, patients with MUS shared how a FSG would provide an opportunity for connection to others with MUS (i.e., peers), support and validation. A collaborative Working Group, including patients with lived experience with MUS, social workers with FSG experience, a primary care physician and pilot project team members, was then formed to develop the structure and content for the FSG. Feedback from the patient engagement sessions was incorporated into the design and content of the FSG (e.g., improving communication with PCPs and learning coping strategies). Each session was structured to include participant rounding (e.g., checking in with each other), goal setting and tracking (e.g., short-term health goals to manage MUS) using a symptom tracking booklet (aka pocketbook), a mindfulness activity (e.g., breathing exercises), and a facilitator-led skills development (see Table 1 for weekly topics).

**Table 1:** Facilitated Support Group Weekly Topics^a^

Week	Topics	Focus of Weekly Content
1	Introductions and Establish Goals of the Group	Introductions and establishment of group norms and expectations of participants to create a safe environment
2	Understanding where you’re at	Documenting changes in symptoms, mood and function and importance of self-reflection
3	Getting the most from your medical appointment	Strategies to improve patient-provider communication and making the most of medical appointments
4	Understanding the Brain-Body Connection	The mind–body connection and the impact on health and well-being
5	Reducing Stress	Stress reducing strategies, including physical activity, healthy eating, sleep and relaxation techniques
6	Living with Stress	Techniques and approaches to help manage the ongoing stress of living with MUS
7	Driving your Care	Perspectives and insights on the healthcare system and how to navigate its complexity
8	Topic determined by the group	Example: Life hacks for people with chronic conditions
9	Topic determined by the group	Example: The science behind stress
10	Wrap up	Reflection about the weekly sessions and discussion about the post-FSG evaluation

^a^Weekly agenda: Welcome, participant rounding, facilitator-led skills development (weekly topics described above), goal-setting for subsequent week (using the pocketbook), wrap-up

#### FSG referrals

The pilot project team shared an information poster and pamphlet describing the pilot FSG with PCP practices within the local community. PCPs referred patients to the FSG if they were between the ages of 18–64 years and experiencing MUS (> 6 months), for which medical examination has not revealed a condition that adequate explains the symptoms, impacting daily functioning and quality of life. Participants were ineligible if they had a significant cognitive impairment or major acute/palliative illness, and/or were unable to attend in person. Facilitators completed intake calls with each participant to review eligibility criteria and enroll participants. Four participants were enrolled in in the first group and five in the second group. One participant in the second group stopped attending the FSG after the first session and did not provide consent to be contacted for an interview. The FSGs each ran for ten weeks consecutively and consisted of weekly, one and a half hour sessions co-led by two experienced facilitators with clinical backgrounds (i.e., social worker or occupational therapist) who had previous experience leading FSGs. Based on patient feedback, the Working Group decided to host the FSG in a community centre in southern Ontario, Canada, rather than a hospital or medical office in an effort to increase patient comfort and openness to share their experiences.

### Study design – qualitative evaluation of facilitated support group

Our qualitative research team conducted an evaluation of the piloted FSGs using a descriptive methodological approach, a naturalistic approach that depicts participants’ experiences with the phenomenon that is under investigation [36, 37]. This methodology is well suited for an exploratory, pragmatic study focused on understanding patient views about physician interactions and how healthcare systems are organized [38, 39]. This approach helped us gain firsthand knowledge of participants’ views about the FSG to improve the design and implementation of subsequent FSGs. The evaluation involved semi-structured interviews with participants and facilitators either in-person or by telephone. Participants and facilitators were interviewed about their experiences with the FSG to learn about: 1) what they thought worked well; 2) what they thought could be improved and; 3) what types of support and resources they think are needed for people with MUS. Information from the first group of interviews was used to inform and improve the content for the second group. For example, the first group mentioned that tracking and providing progress on weekly goals felt like homework; thus in the second group, the facilitators put less emphasis discussing progress and invited the group to share as they felt comfortable. This study was carried out in accordance with relevant guidelines and regulations and was approved by the Trillium Health Partners Research Ethics Board (ID#933).

### Recruitment and data collection

During the first weekly session of each group, facilitators provided participants with an information sheet detailing the qualitative evaluation and obtained written consent to be contacted from those interested (8 participants total) in participating in the evaluation of the FSG. Once the ten week program was completed for each group, a qualitative researcher (MM) contacted participants who gave consent to be contacted and scheduled a one-hour telephone interview. Facilitators were also invited to participate in an interview to describe their experiences with group facilitation and what they observed of the group (e.g., group dynamics, content preferences). All participants provided written informed consent before participating in an interview with a qualitative researcher (MM). Interviews took place within two to four weeks after each FSG’s final session. Semi-structured interview guides (see Additional file 1 and 2, interview guides for participants and facilitators, respectively) were developed by the research team and piloted with the Working Group for length, clarity, and relevance. The same interview guides were used for both FSGs and concentrated on the following key topic areas: 1) participant expectations and experiences 2) perceived impact of participation 3) perceptions of facilitators and/or barriers to implementation. Eleven of the twelve interviews were digitally recorded with consent and professionally transcribed. One participant declined to be recorded, but gave permission for the interviewer to take notes during the interview to use as part of the analysis. Participants received a thank you letter and $25 honorarium for contributing to the evaluation.

### Data analysis

A thematic analysis was used to identify common themes that captured strengths and challenges of the FSG within and across the interview data [40]. Since apriori hypotheses are not engaged in a qualitative descriptive approach [38], the research team was open to the unique insights of participants’ experiences of the FSGs during our analysis [41]. Transcripts were reviewed independently by project team members (EM; MM; JS) and a sample of interviews were coded by the team who met to discuss their independent findings and develop a coding framework. Two team members (MM; JS) then coded the remaining interviews using this framework meeting with the larger qualitative team (EM; MM; JS; CD) to discuss differences in data interpretation. Codes were then combined into broader themes during a series of team meetings where the relationships between the themes were explored and summarized. The team maintained an audit trail of memos, team meetings, analytical questions that arose and decisions that were made to enhance transparency [42]. Data management and analysis was supported using MAXQDA, a specialized qualitative data software program.

## Results

### Overview

A total of 12 people (eight participants and four facilitators) were interviewed (Table 2). The most common long-term (≥ six months) self-reported symptoms participants had been experiencing were related to fatigue (e.g., trouble sleeping, low energy), headaches, chronic pain in the back, chest, or joints, and gastrointestinal symptoms (e.g., nausea, bloating). Other symptoms included, but were not limited to, heart palpitations, blurred vision, dizziness, and shortness of breath. Each facilitator had a clinical background (2 social workers; 2 occupational therapists). Described below are three key themes that were identified through our analysis of the data: 1) feeling validated through sharing similar experiences with peers; 2) learning practical symptom management and coping strategies; and 3) gaining new perspectives for navigating conversations with PCPs.

**Table 2:** Participant Characteristics (*n* = 12)

Characteristics	Patients (*n* = 8)	Facilitators (*n* = 4)
**Age range**		
18–39	4	1
40–79	4	3
**Sex**		
Male	2	1
Female	6	3
**Marital status**		
Single/Divorced/Separated	4	0
Married	4	4
**Highest Level of Education**		
High School	3	0
College graduate	2	0
Undergraduate degree	2	2
Advanced degree	1	2
**Employment**		
Full-time	2	1
Part-time	0	3
Other (e.g., unemployed, student, retired)	6	0

### Feeling validated through sharing similar experiences with peers

Most participants reported that they valued being with others who understood their experience of living with MUS. Although many participants described having a strong support system, they reported that their family and friends could not truly understand their experiences of living with MUS (e.g., living with uncertainty, feelings of frustration, coping with chronic fatigue or pain). For example, when asked why they wanted to join the FSG, one participant mentioned wanting to hear other peoples’ perspectives about living with MUS as a source of encouragement in dealing with uncertainty:

“My expectation is that I would get some support…I would get support to help me go through (this) and in the group, there were others with like similar (experiences). So then I take that as an encouragement to myself and know that although I am alone in my situation but still going through the journey (and) there are others on the same journey.” (Participant #12, G2, female)

Another participant shared how they appreciated having the opportunity to connect with peers living with a condition that is not always viewed as legitimate:

“Well I think I was just frustrated because no one was listening to me, no one was validating what I was going through and then…if there’s other people that are willing to share their experiences, then I think that kind of just gave me a bit of confidence. I’m okay, so I won’t be the odd one out in the room and that just kind of I guess inspired me to join the group.” (Participant #6, G1, female)

Although this participant felt welcomed by other group members, they acknowledged that it was difficult to find common connections because they were at a different life stage:

“I was the youngest person in the group, there was a significant age gap between me and the other members…obviously, they were really understanding but they couldn’t really help me in a way because they were all I guess either working or some of them are married or whatever…So it was I felt like they could kind of connect more than I could.” (Participant #6, G1, female)

### Learning practical symptom management and coping strategies

When asked to reflect on what they thought about the weekly content, participants mentioned how useful it was to learn practical approaches to manage daily living tasks such as sleep, physical activity, nutrition, and stress relief techniques. For example, the facilitators observed that participants enjoyed learning practical tips and tricks to help complete daily tasks that can be challenging when experiencing symptoms, such as chronic fatigue or pain. One facilitator reflected on the groups’ response to watching a demonstration use of a stocking aid:

**“**(Facilitator’s name) brought in – we called in the ‘sock-putter-onner’. It’s the device that helps people put their socks on, and we made a joke of it, but everyone’s like, “That’s so cool and that’s so awesome.” Like just when you’ve got back pain, when you’ve got arthritis, so things are generalizable to some degree.” (Facilitator #8, G2, female)

One participant described how a meditative body scan activity, led by facilitators, helped them to visualize where they felt pain and then reflect upon what factors could be further exacerbating the pain:

“I think for example me, I was like able to think back, “I’m like okay, so the pain is in my lower back, what am I doing that it’s over there, right?” So putting it on paper really sort of just like makes it more clear for you to see like okay, what can you change.” (Participant #6, G1, female)

While learning practical strategies were useful for participants, other aspects of the FSG were underutilized. For example, one facilitator observed how the pocketbook’s appeal diminished over the course of the weekly sessions:

“I think things around like the goal setting, was a little bit sort of elementary for them. I think, most of them had a good understanding of how to set goals… The pocketbook, to some extent, was less useful for them. I think they were definitely open to trying it. And, some of them did use it for a few weeks. I feel like that sort of fizzled out a little bit towards the end.” (Facilitator #1, G1, male)

Participants appreciated that the facilitators had clinical backgrounds; however, they wished for more in-depth input and clinical expertise from additional allied healthcare providers:

“Having actual scheduled certain professionals coming in to kind of discuss or maybe to have the opportunity to bounce some questions off of them, outside of the facilitators themselves…A dietitian, somebody to speak on sleep hygiene, and someone to speak towards the physical aspect (physiotherapy, occupational therapy), those three for me, personally, would have been helpful.” (Participant #9, G2, female)

Although the majority of participants enjoyed learning coping strategies, one participant felt the FSG had nothing new to offer them above and beyond their own research into available resources for living with unexplained chronic fatigue and pain:

“I felt like I wasn’t getting too much from the group because I've already done my own, you know, like therapy and kind of acceptance, and how I know all of the resources available and I've utilized a lot of them. So, for me it was kind of like well I'm giving up an evening that I could be resting or spending time with my husband.” (Participant #11, G2, female)

### Gaining new perspectives for navigating conversations with PCPs

When asked what weekly sessions participants enjoyed the most, participants frequently spoke about how relevant they found weekly sessions offering insights into the healthcare system. In particular, participants valued the session that involved an in-depth conversation with a PCP. Participants were interested in learning about a ‘day in a life’ of a PCP and how PCPs interact with patients with MUS. This information was useful for participants because in addition to learning how to cope with MUS, they are also trying to cope with a complex and siloed system that is not built well to handle the uncertainty of MUS. One participant reflected on how this was a unique opportunity for the group, as time limited medical consults provide little opportunity to ask PCPs questions beyond the purpose of the consult:

“It seemed like everyone was engaged in the family doctor (session) and I think honestly that's because none of us could get access to just sitting with a family doctor and like picking their brain and understanding the healthcare system and the doctor perspective. None of us could get that because when we go see our family doctor it's a 15 minute conversation.” (Participant #3, G1, female)

Although participants appreciated learning about the PCP role in the context of the healthcare system, one facilitator observed that the conversation could have focused more on how patients and PCPs can navigate the healthcare system together more effectively:

“I felt that there was a little bit of frustration after that [session]; not that it wasn’t viable to have the chat, but perhaps it could be like navigating your care, understanding things from this side of the bedside.” (Facilitator #8, G2, female)

A session led by the facilitators that provided information to help participants prepare for future medical appointments and help guide conversations with their PCP was also well received. One participant shared how they felt more confident about effectively communicating their needs to their PCP and hopeful that future interactions would be more positive:

“I feel like that’s what I really learned in the group, like that the doctor’s not your enemy sort of, like you can go in and say like your biggest problem and they shouldn’t judge you. Because I feel like throughout my experience with the (past) two years is like I have lost faith in doctors. Right? So I would go in and I wouldn’t even mention my biggest concern. So I feel like this group kind of helped me realize like it’s okay, you need to tell them what you’re going through -- but be prepared, like have a list of symptoms, like have your facts straight, basically.” (Participant #6, G1, female)

## Discussion and conclusion

### Discussion

This paper provides insights about participant and facilitator perceptions of a FSG for patients with MUS. Participants cited that one of the biggest impacts of the FSG was feeling validated by others who could relate to their situation. Our findings support the principle that people who deal with constant health-related stressors find unique value in receiving support from peers who are empathetic and non-judgemental [43]. This is important, as society is quick to question the legitimacy of an undiagnosed illness [33]. Patients with MUS describe feeling stigmatized [44] and having their illness dismissed by PCPs, family, and friends [44–47]. Many patients with MUS struggle with their identity (i.e., feeling like a different person as a result of the illness) while making sense of their illness [8, 48] and hiding their symptoms to avoid being embarrassed when a condition cannot be validated with an established medical diagnosis [9].

Similar to our findings, when given an opportunity to connect with peers, Kornelsen et al. [9] described that patients with MUS valued engaging with others who were also grappling with the uncertainty of an undiagnosed illness/symptoms. These patients also found value in reading books and visiting websites where people described their struggles living with MUS [9]. Our evaluation of the FSG highlights how critical creating a supportive environment and forum was for individuals to feel comfortable and respected when sharing their experiences. We suggest the inclusion of peer support when developing resources for patients with MUS to help them better understand, manage, and make sense of their illness. Additionally, our findings show the importance for some participants to connect with others in a similar life stage who may be facing similar challenges coping with MUS and should be an additional consideration if possible when organizing FSGs.

Participants also appreciated learning lifestyle or behavioural coping mechanisms to support their overall health and well-being. Patients with MUS have reported coping with their unexplained illness in various ways, such as diverting their focus away from symptoms (e.g., reading a book) [47], taking control over their body by engaging in self-care (e.g., yoga, walks) [8], or modifying lifestyle behaviours to improve overall health (e.g., being more active, quitting smoking) [9]. In a recent review by van Gils et al. [49], self-help interventions were found to be associated with a decrease in severity of symptoms and increase in quality of life for various types of MUS [49]. Creating tools and resources for patients living with MUS may contribute to a better quality of life and a more positive outlook when dealing with an unpredictable health situation. However, when dealing with patients who have long-term conditions, it is important to recognize that focusing on the medical aspects of self-management and coping should not overshadow other types of assistance that are highly valued by patients in group programs, such as connection to peers and re-building diminished support networks [28]. Furthermore, while the facilitators had social work and occupational therapist skillsets, participants wanted more interactions with allied health professionals during weekly sessions (e.g., dietitian to discuss food and health). Gol et al. [50] reported that PCPs often share symptom management strategies and recommendations with patients with MUS that are unclear, ambiguous and not relevant [50]. As a result, patients with MUS may be seeking support beyond what their PCP can offer during a typical time limited consult to understand and manage their illness.

Learning about how to better navigate the healthcare system and interact with physicians particularly resonated with participants. The positive responses to sessions offering insights into the PCP role and the healthcare system identifies a disconnect in how patients understand physician approaches to care for patients with MUS. Participants described feeling hopeful that they would have more positive interactions with their own PCP and be more proactive in structuring conversations around their care moving forward. Our findings suggest that providing education and tools may help patients feel more empowered and confident in managing their care when interacting with PCPs. However, it is important to recognize that while some patients with MUS accept being in the driver’s seat of their own care [9, 47], others do not embrace this responsibility and view the traditional patient-provider relationship as a lifeline in the face of uncertainty [9].

Bridging that gap of understanding also requires physicians to reconsider their communication and interactions with patients with MUS. Johansen & Risor [22] suggest that PCPs attempts to expand consultation approaches can be limited, particularly when “struggling to use explanatory models and a bio-psychosocial approach that often lack patient-centeredness and does not transform into shared epistemology” [22]. In general, physicians receive little or no training about how to investigate and manage MUS [23] and often rely on anecdotal clinical experience and personal judgement [23, 51]. A lack in a consistent approach to how physicians navigate MUS may result in patients with MUS receiving mixed messages and negatively affect the patient-provider relationship [23]. From the patient perspective, there is a preference that PCPs provide more personalized and individualized care (i.e., paying attention to the individual, conversation, and symptoms) and treat patients as equal partners when discussing care options [16].

#### Strengths and limitations

Our evaluation exploring the strengths and challenges of a MUS FSG pilot project was limited in scope, as it involved a single setting intervention with limited time, resources and small sample size. Although limited in scope, by engaging a qualitative descriptive approach we were able to gain firsthand knowledge of participants’ experiences of the FSG [36, 38] and provide insights into how a FSG developed for patients living with MUS can be a resource for this patient population. In future studies it would be beneficial to present our results to participants (i.e., member checking) to check for accuracy and resonance with their experiences [52]. Our sample did not capture diversity across categories such as race, ethnicity, sexual identity and socioeconomic status; but given the study’s exploratory design we feel that the sample was adequate for the purpose of the study [53]. Future research should include a larger sample size using maximum variation sampling to ensure a more diverse sample and provide more insights to support our conclusions. Our findings do not include the perspectives of patients with MUS who may have been too sick or unable to enroll in person for the FSG. Future support groups might consider the addition of an online as well as in-person attendance option [54]. Last, more rigorous evaluations (e.g., using mixed methods approaches) are needed to determine if a FSG provides any long lasting impact, such as reduced consultations with PCPs and referrals to other care professionals, and improvement in quality of life. Despite these limitations, we believe our findings build on recent literature calling for a shift in approaches away from diagnosis-focused care to more chronic health self-management approaches.

### Conclusion

Creating a space for participants with MUS to share and reflect on their experiences together was highly valued by participants enrolled in a FSG. Providing educational tools and resources to help patients cope with symptoms and improve communication with PCPs made participants feel more hopeful about managing MUS moving forward. The results of this evaluation study complement current literature calling for a flexible approach to the management of MUS that considers both the biomedical and humanistic perspective [55, 56]. Our findings also support the integration of other types of health care professionals and/or peers to support patient learning and sustainable care that is driven by the needs of this patient population [8].

### Practice implications

Our evaluation highlights that additional support offered by a FSG enhances care well beyond the frustratingly limited support that many patients with MUS feel that the health care system offers (32). These findings are relevant to PCPs and multidisciplinary group practices, medical educators and health service planners, among others, as there is a need to expand patient care and delivery of self-management strategies for patients who suffer from long-term unexplained symptoms. We suggest that future research explore how to better engage and train health care professionals to support patients in coping with MUS and improve their quality of life. Furthermore, additional engagement and co-design with patients with MUS should explore reasons that patients may not participate or withdraw from support groups and identify meaningful outcomes for patients with MUS participating in professionally and/or peer support guided programs.

## Supplementary Information

**Additional file 1:** Semi-structured interview guide for patients.

**Additional file 2:** Semi-structured interview guide for group facilitators.

## Data Availability

The data that support the findings of this study are available on request from the corresponding author (M.M.). The data are not publicly available as the data contains information that could compromise research participant privacy or consent.

## Abbreviations

MUS: Medically unexplained symptoms
FSG: Facilitated support group
PCP: Primary care physician
MPA: Medical Psychiatry Alliance
