# Supplementary material for: Making sense of symptoms, clinicians and systems: a qualitative evaluation of a facilitated support group for patients with medically unexplained symptoms
Source: BMC Fam Pract. 2021 Jul 1;22:142. doi: 10.1186/s12875-021-01495-9 (PMC8252243; doi:10.1186/s12875-021-01495-9)
Supplement: Supplementary file 1 — Additional file 1: Semi-structured interview guide for patients. [file 12875_2021_1495_MOESM1_ESM.doc]

**Additional file 1: Semi-structured Interview Guide for Patients**

*This script will be used before each patient interview to explain the purpose of the interview, review key points from the informed consent form, and provide an opportunity for the participant to ask questions.* *Please note that these guides only represent the main themes to be discussed with the participants and as such do not include all the various prompts that may also be used. Non-leading prompts will also be used, such as “Can you please tell me a little bit more about that?” and “What does that look like for you?” We will also ask for concrete examples of general descriptive statements that are made.*

**Introduction:** Thank you for agreeing to participate in this interview. The interview, which is really more like a conversation, should take about one hour. There are no right or wrong answers. We are interested in hearing about your experiences with this project, which will help inform us about how we should proceed with developing and supports and resources for patients living with MUS. I would like to remind you that participation in this interview is voluntary. You can also withdraw from this study at any time; contact information to do so is provided in your study information letter and informed consent package.

With your permission, I would like to audio record the interview because I don’t want to miss any of your comments; however, you may ask me to turn off the digital recorder at any time during the interview. All your responses will be kept confidential. This means that any identifying information will be removed from your interview responses, which will only be shared with the project team members. We will also make sure that any information we include in our report does not identify you as the respondent. Are there any questions about what I have just explained? May I turn on the digital recorder?

1. Background Information

*We are interested in learning about you and how you came to participate in the project.*

- Can you tell me about how you became interested in joining a facilitated support group for patients living with MUS?

1. Introduction to the Project

*We would like to learn about your initial experiences with the project.*

- Tell me about the first time you heard about the facilitated support group being offered?
  - Prompt: What were your initial expectations?
  - Prompt: How did you decide that joining a support group was right for you?
- What did you think about the initial assessment and screening process?
- Tell me about the first few group sessions.
  - Prompt: What stood out to you?

1. Perceptions of project components and impact on participants

*We are interested in learning more about your specific experiences with this project and how it may impact care for patients living with MUS.*

- What stood out for you as something you have enjoyed about the program? Can you give an example?
- What stood out for you as something that could be improved with the program? Can you give an example?
- What are your thoughts about the amount of time you spent on group activities?
  - Prompt: Tell me what you thought about these activities were organized.
- What are your thoughts about the time you spent interacting with other group members?
  - Prompt: Tell me about what you learned from others in the group; length of time for free discussion
- Tell me what you think about the content that was delivered by the facilitator.
  - Prompt: What information did you find the most useful? Least useful?
  - Prompt: What is an important topic that you were hoping to have learned that wasn’t covered in the sessions?
- Tell me what you think about the activities that were planned for the sessions.
  - Prompt: What activities did you find the most useful? Least useful?
  - Prompt: Are there any other types of activities you would have hoped would be included in the sessions?
- In what ways have things changed for you since joining the project?
  - Prompt: Are there any specific elements that you learned in group that you continue to use?
  - Prompt: In what ways have things stayed the same?

1. Interactions between patients and health care professionals

*We are interested in learning more about typical interactions with the group facilitators, as well as how your involvement in the support group has impacted interactions with your family physician.*

*With respect to your interactions with the group facilitator(s):*

- Tell me what you thought about the group facilitator(s).
  - Prompt: How did the facilitator(s) communicate with the group?
- How would you describe the role of the facilitator(s)?
  - Prompt: E.g., provide education, moderate discussions, give advice

*With respect to your interactions with your family physician:*

- Tell me about your health since joining the project.
  - Prompt: Did you notice any change in physical or non-physical symptoms?
- How has your involvement in the facilitated support group impacted your care?
  - Prompt: Tell me about something you learned that has helped with the management of your symptoms.
- How, if at all, did this project change the way in which your symptoms are managed?
  - Prompt: E.g., Self-management of symptoms? Shared decision-making with PCP regarding care planning process? Communications with PCP?

1. Experiences with project education/resources and training

*We would like to know more about your experiences with the project in learning about your health challenges and how to manage them.*

- How, if at all, did the project change your knowledge about your condition?
  - Prompts: Changes in practice, behaviour, self-management
- What kinds of additional information and education would you find helpful?

1. Project implementation

*We are interested in learning more about your thoughts about what aspects of the project support program could be improved.*

- In your opinion, how many weeks should the facilitated support group be held?
  - Prompt: More or less than current during of 8 weeks?
- In your opinion, what other topics would you like to have covered?
- If you had a wish list for things that could be improved, what would be on that list?
- Is there anything else you would like to add that hasn’t been mentioned so far?
- What advice would you offer to someone who was interested in joining a support group for patients living with MUS?

**Thank you for your feedback!**
