# Supplementary material for: Making sense of symptoms, clinicians and systems: a qualitative evaluation of a facilitated support group for patients with medically unexplained symptoms
Source: BMC Fam Pract. 2021 Jul 1;22:142. doi: 10.1186/s12875-021-01495-9 (PMC8252243; doi:10.1186/s12875-021-01495-9)
Supplement: Supplementary file 2 — Additional file 2: Semi-structured interview guide for group facilitators. [file 12875_2021_1495_MOESM2_ESM.doc]

**Additional file 2: Semi-structured Interview Guide for Group Facilitators**

*This script will be used before each group facilitator interview to explain the purpose of the interview, review key points from the informed consent form, and provide an opportunity for the participant to ask questions.* *Please note that these guides only represent the main themes to be discussed with the participants and as such do not include all the various prompts that may also be used. Non-leading prompts will also be used, such as “Can you please tell me a little bit more about that?” and “What does that look like for you?” We will also ask for concrete examples of general descriptive statements that are made.*

**Introduction:** Thank you for agreeing to participate in this interview. We are interested in hearing about your experiences with this project, which will help inform us about how we should proceed with developing supports and resources for patients living with MUS. The interview, which is really more like a conversation, should take approximately an hour. With your permission, I would like to audio record the interview because I don’t want to miss any of your comments; however, you may ask me to turn off the recorder at any point throughout the interview. All responses will be kept confidential. This means that any identifying information will be removed from your interview responses, which will only be shared with the project team members. We will also make sure that any information we include in our report does not identify you as the respondent.

I’d like to remind you that participation in this interview is voluntary and your decision to participate or not, will not affect you professionally. You do not have to answer any question(s) that make you feel uncomfortable and you can stop the interview at any time and for any reason. I want to hear what you have to say and there are no right or wrong answers to any of the questions. Are there any questions about what I have just explained? May I turn on the digital recorder?

1. Background Information

*We are interested in learning about your thoughts of this project.*

- Tell me how you came to be involved in this project?
- What made you interested in facilitating a support group for patients living with MUS?

1. Introduction to the Project

*We would like to learn about how you manage the screening process and your overall thoughts about the screening process.*

- Describe the process of screening patients for this group support.
  - Prompt: In your opinion, what did the patients think about the screening process?
- What, if anything, would you change about the screening process?
  - Prompt: What are your thoughts about the eligibility criteria?

1. Perceptions of project components and impact on participants

*We are interested in learning more about your specific experiences with this project and how it may impact patients living with MUS.*

- Tell me what you think about the intended program goals and objectives?
  - Prompt: Do you think the goals and expectations set for the program were met? Why or why not?
- What are your thoughts about the amount of time spent on the semi-structured components of the group sessions?
  - Prompt: Based on your observations, tell me what you think worked well and what could be improved?
- What are your thoughts about the amount of time group members were given to interact with each other?
  - Prompt: Tell me about how the group interacted with one another.
- Tell me what you think about the content designed for the group sessions.
  - Prompt: What information do you think was the most useful for group members? Least useful?
  - Prompt: From your perspective, are there any topics that were not covered during these sessions that you think should have been?
- Tell me what you think about the activities that were planned for the sessions.
  - Prompt: What activities do you think were the most useful for group members? Least useful?
  - Prompt: Are there any other types of activities that you would like to implement in future group sessions?
- In your opinion, how do you think group members benefited from the facilitated support groups?
  - Prompt: E.g., learning from others, increased confidence about upcoming medical visits

1. Interactions between patients and group facilitators

*We are interested in learning more about your perceptions about the interactions you had with patients throughout the program?*

- Tell me about how you viewed your role as a facilitator?
  - Prompt: E.g., Provide education? Moderate discussions? Ensure program outcomes were met? Did you complete any training and/or preparation for this role
- Tell me about a time during sessions where you found facilitating challenging?
  - Prompt: Are there any resources or training that would have made you better prepared in this situation?
  - Prompt: Tell me about your interactions with the co-facilitator.

1. Experiences with project education/resources and/or training

*We are interested in learning more about your thoughts about the types of supports and resources that should be made available to patients and facilitators in this project.*

- Are there any supports or resources that would help you better facilitate group sessions for patients living with MUS?
  - Prompt: What kind of training opportunities would you like to see available for facilitators working with this population?
- Are there any supports or resources you think that patient group members could have benefited from that were not offered in the project?
  - Prompt: Do you think there should be opportunities for patients to connect outside of the group with the facilitators and/or other group members?

1. Project implementation

*We are interested in learning more about your thoughts about what aspects of the program could be improved.*

- In your opinion, for how many weeks should the facilitated support group be held?
  - Prompt: More or less than current duration of 8 weeks?
- In your opinion, do you think the facilitated support groups should have more or less structured components?
  - Prompt: Based on your observations, would group members benefit from having more or less time to work with their peers to apply what they have learned in the sessions?
- If you had a wish list for things that could be improved, what would be on that list?
- Is there anything else you would like to add that hasn’t been mentioned so far?

**Thank you for your feedback!**
